# SPANG: a SPARQL client supporting generation and reuse of queries for distributed RDF databases

**DOI:** 10.1186/s12859-017-1531-1

**Published:** 2017-02-08

**Authors:** Hirokazu Chiba, Ikuo Uchiyama

**Affiliations:** National Institute for Basic Biology, National Institutes of Natural Sciences, Nishigonaka 38, Myodaiji, Okazaki, Aichi 444-8585 Japan

**Keywords:** Semantic Web, SPARQL, RDF, Database integration, Unix command

## Abstract

**Background:**

Toward improved interoperability of distributed biological databases, an increasing number of datasets have been published in the standardized Resource Description Framework (RDF). Although the powerful SPARQL Protocol and RDF Query Language (SPARQL) provides a basis for exploiting RDF databases, writing SPARQL code is burdensome for users including bioinformaticians. Thus, an easy-to-use interface is necessary.

**Results:**

We developed SPANG, a SPARQL client that has unique features for querying RDF datasets. SPANG dynamically generates typical SPARQL queries according to specified arguments. It can also call SPARQL template libraries constructed in a local system or published on the Web. Further, it enables combinatorial execution of multiple queries, each with a distinct target database. These features facilitate easy and effective access to RDF datasets and integrative analysis of distributed data.

**Conclusions:**

SPANG helps users to exploit RDF datasets by generation and reuse of SPARQL queries through a simple interface. This client will enhance integrative exploitation of biological RDF datasets distributed across the Web. This software package is freely available at http://purl.org/net/spang.

**Electronic supplementary material:**

The online version of this article (doi:10.1186/s12859-017-1531-1) contains supplementary material, which is available to authorized users.

## Background

Because of advances in biotechnologies, various types of biological data have drastically increased in the past decade. Because of the volume, heterogeneity, and continual growth of biological data, it has become increasingly difficult for individual researchers to manage an entire dataset in a single repository. In this context, Semantic Web technology [1] has attracted attention as a promising approach of knowledge management [2]. In the Semantic Web, all information is described in the Resource Description Framework (RDF) [3], in which every piece of information is in the form of a triple containing a subject, predicate, and object and each resource is represented by a Uniform Resource Identifier (URI). The RDF works as a general framework of knowledge representation and the URI assures valid integration of data collected from different sources. Furthermore, knowledge extraction from the RDF can be implemented using a powerful query language called the SPARQL Protocol and RDF Query Language (SPARQL) [4]. SPARQL specifications include federated query functionality [5], by which distributed databases can be queried in an integrative manner. Thus, Semantic Web technology provides a basis for flexible integration of the increasing amount of heterogeneous data. In fact, many biological databases have already adopted the Semantic Web [6–9].

Despite the well-designed basis of Semantic Web technologies, several obstacles that prevent users including bioinformaticians from utilizing RDF databases still remain. The main hurdle for most users is writing SPARQL, which often includes cumbersome coding tasks. For example, SPARQL permits inclusion of subqueries for distinct endpoints in a federated query; however, writing such a nested query is a complicated task and can be a technical obstacle for most users. Several approaches for supporting SPARQL coding currently exist. Examples include SPARQL editors with useful functionalities such as URI autocompletion [10], and graphical support for step-by-step construction of SPARQL queries [11, 12]. Despite these approaches, constructing executable SPARQL code, even for a simple query, still remains a time-consuming task; thus, a mechanism that saves time of preparing SPARQL code is necessary to maximize the use of available RDF datasets. As an alternative approach to this issue, a wiki-based portal for sharing SPARQL queries was constructed [13], which can bypass the burdensome coding task. Although the queries registered on this service can be executed on the portal site, a mechanism for reusing these queries in other environments would maximize the usefulness of the accumulated queries.

Here, we developed SPANG, a client that supports querying by generation and reuse of SPARQL codes through a simple interface. Taking advantage of the common “triple” form of RDF data, SPANG generates typical queries without the need for SPARQL coding. Even in complicated queries, SPANG can construct runtime queries using predefined templates. Regarding the federated query, SPANG realizes a similar functionality by combining multiple queries through a Unix pipe. SPANG, with its unique features, minimizes the burden of coding SPARQL, thereby enhancing integrative exploitation of distributed databases.

## Implementation

The SPANG package includes the main spang command, which can be used in the Unix command-line environment. In general, the spang command helps users to query RDF databases by dynamically generating SPARQL queries according to the supplied command-line options or arguments. More specifically, spang has two execution modes:

1. Shortcut mode, in which users need only specify command-line options to generate a simple query. Specific command-line options, including -S *SUBJECT*, -P *PREDICATE*, -O *OBJECT*, -L *LIMIT*, and other modifiers, are interpreted as shortcuts for generating typical SPARQL queries (see Additional file 1).
2. Template mode, in which users can generate a query using a SPARQL template and parameters. The template can be either a local file or a remote file published on the Web. The specified parameters replace the placeholders included in the template to generate a runtime query.

Although each spang process submits a query to a specified database, the spang process can be combined with other Unix processes through a Unix pipe. Notably, multiple spang commands, each with distinct target database, can be combined through a Unix pipe by transferring variable bindings between queries, thereby realizing federated use of multiple databases.

The SPANG package is implemented in Perl. Specifically, the spang command accesses remote SPARQL endpoints using the Perl LWP module. To lower the initial hurdle of querying with SPARQL, the SPANG package provides predefined configurations, including i) nicknames for SPARQL endpoints, ii) frequently used prefix declarations for URIs, and iii) SPARQL template libraries. Furthermore, users can extend the configurations by preparing user-defined configuration files.

## Results

### Simple queries using SPARQL shortcuts

**Fig. 1:**
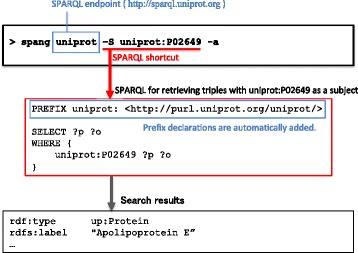
Example usage of SPANG with SPARQL shortcuts. The command line accesses the UniProt SPARQL endpoint and searches for triples that have uniprot:P02649 as a subject

SPANG can generate and execute simple queries by specifying a set of SPARQL shortcuts and additional options. An example of such queries is,

- spang uniprot -S uniprot:P02649 -a

where the first argument is the target SPARQL endpoint and the ensuing arguments are SPARQL shortcuts and an option. The uniprot in the first argument is a predefined nickname for the UniProt SPARQL endpoint [14]. The SPARQL endpoint can be specified in a URL or in a nickname for simplicity. The uniprot: in the third argument is a prefix for URIs of UniProt entries. This example command line searches the UniProt database for statements that have the specified entry ID as a subject (Fig. 1). Using the -a option transforms the URIs in the search result into abbreviated forms using predefined prefix declarations. For example, a URI < http://www.w3.org/2000/01/rdf-schema#label > is transformed into rdfs:label. The result is output to the standard output in the form of tab-separated values by default, as it is suitable for processing by line-oriented Unix programs. In addition, a combination of subject, predicate, and object is possible according to the following:

- spang uniprot -S uniprot:P02649 -P up:organism

where the predicate up:organism is specified to confine the results to organism information. Instead of specifying a predicate, a property path can be used as follows:

- spang uniprot -S uniprot:P02649 -P up:organism/up:scientificName

which retrieves the scientific name of the organism. Thus, the shortcut mode can be typically used to retrieve resources that are associated with a specific subject via arbitrary predicates. More generally, the shortcut mode can generate a SPARQL code containing a certain triple pattern (see Additional file 1). Adding -q option to the command line outputs the generated SPARQL query without executing it, thereby allowing inspection of the internal operation. For the full list of available command-line options, simply type the command spang.

### Using SPARQL templates with parameters

**Fig. 2:**
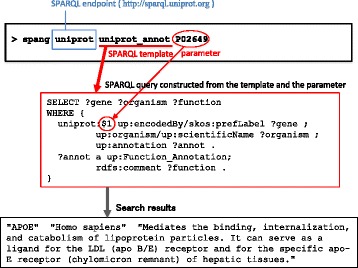
Example usage of SPANG with SPARQL template and parameter. The specified parameter replaces the placeholder included in the template before execution. This query retrieves annotation for the protein P02649 from the UniProt database

Although the SPARQL shortcuts are useful for generating simple queries, they do not cover a complicated query that contains combinations of triple patterns. Thus, SPANG provides a mechanism to generate arbitrary query patterns using SPARQL templates. An example of such is,

- spang uniprot uniprot_annot P02649

where the first argument is the target SPARQL endpoint, the second argument is the name of the SPARQL template, and the ensuing argument is the parameter of the template. The specified parameter replaces the placeholder (represented as $1) included in the template before execution (Fig. 2). uniprot_annot is the name of a SPARQL template included in the predefined SPARQL library and P02649 is a parameter. This example query retrieves annotation for a protein P02649 from the UniProt database.

Whereas the templates usually assume specific target databases, some templates are generally applicable to any SPARQL endpoint; for example,

- spang uniprot regex_class’^apolipoprotein’

where regex_class is a SPARQL template to search for specific classes matching a given pattern of regular expression (see Additional file 2 for the SPARQL code). Although this query is submitted to the UniProt database in the example command line, the template can also be used to search other databases (see the practical use case of SPANG given below).

Available SPARQL templates are not limited to the local library. When SPARQL libraries are published on the Web, users can call the templates by means of URIs across the Web. We have prepared a SPARQL template library for the Microbial Genome Database (MBGD) [15], which is available at http://mbgd.genome.ad.jp/sparql/library/. This library can be utilized in a command line such as

- spang mbgd mbgdl:get_ortholog K9Z723

where mbgd is the MBGD SPARQL endpoint [9] and mbgdl: is a prefix for abbreviating the URI of the template get_ortholog in the MBGD SPARQL library (see Additional file 2 for the code). The template can be specified in the full URI or in abbreviated form using the predefined prefix declarations. This example query searches the MBGD database for the orthologs of the specified protein K9Z723 (Photosystem II lipoprotein Psb27).

### Combinatorial execution of multiple queries

**Fig. 3:**
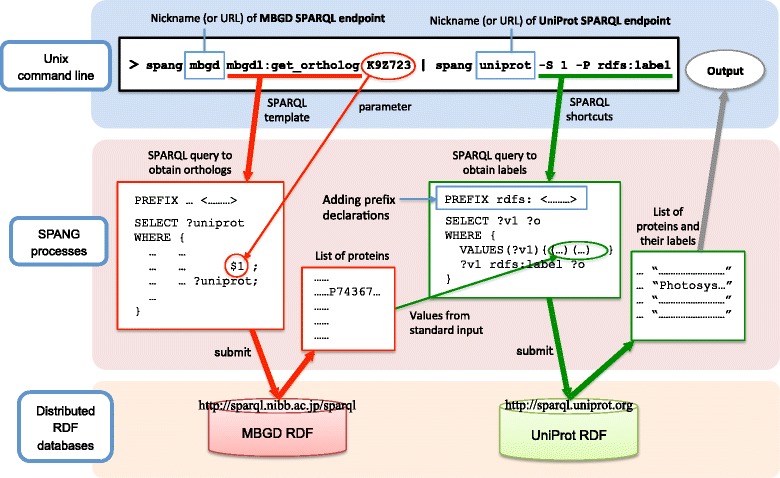
Example command line for executing combinatorial query using SPANG. The command line accesses two databases: MBGD and UniProt. Combinatorial querying against multiple databases is achieved by connecting queries through a Unix pipe. The specified commands first search MBGD for orthologs of K9Z723 and then search UniProt for their protein annotations. mbgdl:get_ortholog is a SPARQL template to obtain ortholog members of a given UniProt ID (see Additional file 2 for the code). The placeholder $1 in the SPARQL template is dynamically replaced by the specified parameter. The command-line option -S 1 is used to set the values from the first column of the standard input as subject

In federated use of multiple databases, SPANG can connect queries for distinct target databases through a Unix pipe. Combining a spang command in shortcut mode and another one in template mode is also possible. An example of such a combination is,

- spang mbgd mbgdl:get_ortholog K9Z723 | spang uniprot -S 1 -P rdfs:label

where the first spang command is the same as the one presented in the previous subsection to search the MBGD database for orthologs of the protein K9Z723; the obtained list of proteins are used in the second command to search the UniProt database for annotations of the given list of proteins (Fig. 3). The option -S 1 is used to specify the values in the first column of the standard input as subject. This combinatorial query enables integrative use of two databases distributed across the Web. Note that the output of the first command can also be used in a different query by altering the second command; for example,

- spang mbgd mbgdl:get_ortholog K9Z723 | spang uniprot uniprot_xref PDB

where uniprot_xref is a SPARQL template (see Additional file 2 for the code), which retrieves cross-references from the UniProt IDs given in the standard input to the database specified as the parameter (in this example, PDB). This example command line searches for entries in the Protein Data Bank (PDB) [16] among orthologs of K9Z723.

### Practical use case of SPANG

**Fig. 4:**
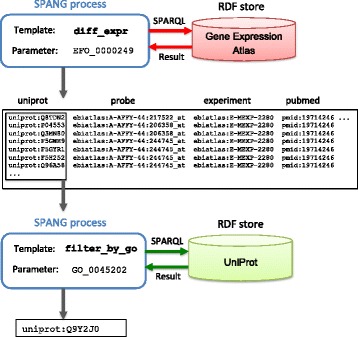
Stepwise execution of SPANG commands for integrative use of RDF databases. The first SPANG command retrieves differentially expressed genes in samples of “Alzheimer’s disease” (EFO_0000249). The second command filters resulting proteins by GO annotation of “synapse” (GO_0045202)

A series of queries that represents a practical use case of SPANG is described below. Suppose that we are examining Alzheimer’s disease by exploring genes associated with it. An important task would be to search for differentially expressed genes in Alzheimer’s disease patients. Differential gene expression data are available from the Gene Expression Atlas [17] constructed on the basis of a variety of samples that are curated and annotated with the Experimental Factor Ontology (EFO) [18]. Given that we do not know specific resource IDs in advance, we would begin the search with a specific keyword. The following query is available to search for relevant resources using a regular expression:

- spang atlas regex_class’^alzheimer’

where atlas represents the SPARQL endpoint for Gene Expression Atlas [7]. This example query gives us a term, EFO_0000249 (Alzheimer’s disease) that is defined in the EFO. The following command line can be used to obtain detailed information about the term:

- spang atlas -S efo:EFO_0000249 -a

which retrieves statements that have efo:EFO_0000249 as a subject. Figure 4 illustrates the following stepwise execution of SPANG. The command line shown below retrieves differentially expressed genes in samples of Alzheimer’s disease and saves the result as a file:

- spang atlas diff_expr EFO_0000249 > result

where diff_expr is a SPARQL template to search for differentially expressed genes specifying a condition of samples (in this example, Alzheimer’s disease). The result includes microarray probes showing signals of differential gene expression, cross-references from these probes to UniProt IDs, and the PubMed entries describing these experiments. In this particular example, the result is derived from a specific microarray experiment [19]. The obtained result can be further processed by other commands; the next command line extracts the first column (protein IDs) and filters them by Gene Ontology annotation [20] to select those related to “synapse” (GO_0045202):

- cut –f1 result | spang uniprot filter_by_go GO_0045202 -a

The result includes the protein Q9Y2J0 (Rabphilin-3A; RPH3A). Recently, it was experimentally shown that reduction of rabphilin-3A in Alzheimer’s disease correlates with dementia severity and amyloid beta accumulation [21]. Thus, stepwise execution of SPANG commands is a useful approach for RDF data integration and knowledge discovery.

All examples of SPANG commands used in this paper are summarized in a table, where they are compared with the corresponding plain SPARQL queries (Additional file 3). It shows that the burden of querying with SPARQL can be reduced by using SPANG commands.

## Discussion

In this paper, we presented SPANG, a SPARQL querying client that has several unique features. First, SPANG provides a shortcut mode that can generate a simple query containing a certain triple pattern. This mode aids querying with SPARQL and is helpful for beginners to start exploring RDF datasets. It is also useful for experienced users of SPARQL, as useful information can often be obtained by retrieving adjacent nodes in RDF graphs using the shortcut mode and efficiently submitting such simple queries is crucial in data mining. Second, for more complicated queries, SPANG provides a template mode, by which existing SPARQL codes can be reused among users. This mode enhances the usage of SPARQL through development of SPARQL template libraries that represent reusable query patterns. The template libraries constructed by experienced users can help other users to efficiently utilize RDF databases. Third, the queries in either shortcut or template mode can be combined in the Unix command line to realize a more complex query. This modular structure of queries has several merits: it reduces complexity of each SPARQL query, leading to easier implementation and debugging of the query; and it extends potential application of each query through combination with other queries or Unix commands.

The predefined SPARQL templates included in the SPANG package are available to help users query some biological RDF databases. However, the range of queries included in the package is limited to rather common ones. The potential use of SPANG can be further extended by database users or database providers through development of SPARQL template libraries. Although a service for sharing SPARQL queries exists [13], it is difficult to execute them directly for instant reuse by users. In SPANG, users can directly call SPARQL templates across the Web. Thus, if an RDF database provider, who knows best the manner in which the database should be used, publishes SPARQL template libraries, database usage can be considerably enhanced. This study suggests the possibility of an open framework of sharing query in a reusable form. Future work may include the standardized use of the query templates, which will further facilitate the sharing of useful queries. Sharing not only data but also queries (i.e., means of interpreting data) on the Semantic Web platform will help the biological research community collaborate in knowledge integration and discovery.

## Conclusions

SPANG enables easy generation of typical queries, thereby reducing the burden of writing SPARQL. SPANG also provides a framework for reusing and sharing arbitrary queries across the Web. Moreover, it enables users to execute complex queries by combining existing query templates. SPANG, with these unique features, facilitates integrative exploitation of published RDF datasets and supports knowledge discovery.

## Additional files

Additional file 1: List of SPARQL shortcuts with example usages. (PDF 71 kb)
Additional file 2: Codes of the SPARQL templates used as examples. (PDF 78 kb)
Additional file 3: Example SPANG commands compared with the corresponding plain SPARQL queries. (PDF 70 kb)

## Abbreviations

EFO: Experimental Factor Ontology
MBGD: Microbial Genome Database for Comparative Analysis
PDB: Protein Data Bank
RDF: Resource Description Framework
SPARQL: SPARQL Protocol and RDF Query Language
URI: Uniform Resource Identifier
