# Supplementary material for: SPANG: a SPARQL client supporting generation and reuse of queries for distributed RDF databases
Source: BMC Bioinformatics. 2017 Feb 8;18:93. doi: 10.1186/s12859-017-1531-1 (PMC5299738; doi:10.1186/s12859-017-1531-1)
Supplement: Additional file 1: — List of SPARQL shortcuts with example usages. (PDF 71 kb) [file 12859_2017_1531_MOESM1_ESM.pdf]

List of SPARQL shortcuts with example usages.

Shortcut	Description	Example Usage	Generated SPARQL pattern
`-S`	Specify a subject	`-S uniprot:K9Z723`	SELECT ?p ?o<br>WHERE {<br>uniprot:K9Z723 ?p ?o<br>}
		`-S uniprot:K9Z723 -f n-triples`	CONSTRUCT {<br>uniprot:K9Z723 ?p ?o<br>}<br>WHERE {<br>uniprot:K9Z723 ?p ?o<br>}
		`-S 1`	SELECT ?v1 ?p ?o<br>WHERE {<br>VALUES (?v1) { $STDIN }<br>?v1 ?p ?o<br>}
`-P`	Specify a predicate	`-P rdfs:label`	SELECT ?s ?o<br>WHERE {<br>?s rdfs:label ?o<br>}
`-O`	Specify an object	`-O tax:9606`	SELECT ?s ?p<br>WHERE {<br>?s ?p tax:9606<br>}
`-L`	Limit outputs	`-L 10`	SELECT ?s ?p ?o<br>WHERE {<br>?s ?p ?o<br>}<br>LIMIT 10
		`-S uniprot:K9Z723 -L 10`	SELECT ?p ?o<br>WHERE {<br>uniprot:K9Z723 ?p ?o<br>}<br>LIMIT 10
`-N`	Output the number of results	`-N`	SELECT COUNT(*)<br>WHERE {<br>?s ?p ?o<br>}
		`-N -O tax:9606`	SELECT COUNT(*)<br>WHERE {<br>?s ?p tax:9606<br>}
`-G`	Output graphs	`-G`	SELECT ?graph<br>WHERE {<br>GRAPH ?graph {<br>?s ?p ?o<br>}<br>}<br>GROUP BY ?graph<br>ORDER BY ?graph
		`-S uniprot:K9Z723 -G`	SELECT ?graph<br>WHERE {<br>GRAPH ?graph {<br>uniprot:K9Z723 ?p ?o<br>}<br>}<br>GROUP BY ?graph<br>ORDER BY ?graph
`-F`	From a specific graph	`-F 'http://sparql.uniprot.org/uniprot/'`	CONSTRUCT {<br>?s ?p ?o<br>}<br>FROM <http://sparql.uniprot.org/uniprot/><br>WHERE {<br>?s ?p ?o<br>}
		`-F 'http://sparql.uniprot.org/uniprot/' -S uniprot:K9Z723`	SELECT ?p ?o<br>FROM <http://sparql.uniprot.org/uniprot/><br>WHERE {<br>uniprot:K9Z723 ?p ?o<br>}
		`-F 'http://sparql.uniprot.org/uniprot/' -N`	SELECT COUNT(*)<br>FROM <http://sparql.uniprot.org/uniprot/><br>WHERE {<br>?s ?p ?o<br>}
