# Supplementary material for: SPANG: a SPARQL client supporting generation and reuse of queries for distributed RDF databases
Source: BMC Bioinformatics. 2017 Feb 8;18:93. doi: 10.1186/s12859-017-1531-1 (PMC5299738; doi:10.1186/s12859-017-1531-1)
Supplement: Additional file 2: — Codes of the SPARQL templates used as examples. (PDF 78 kb) [file 12859_2017_1531_MOESM2_ESM.pdf]

Codes of the SPARQL templates used as examples in the main text.

1) uniprot_annot (included in the SPANG package)

Given a UniProt ID as a parameter, retrieve its gene name, organism name, and functional annotation.

The target database is UniProt.

```
SELECT ?gene ?organism ?function
WHERE {
   uniprot:$1 up:encodedBy/skos:prefLabel ?gene ;
          up:organism/up:scientificName ?organism ;
          up:annotation ?annot .
   ?annot a up:Function_Annotation ;
          rdfs:comment ?function .
}
```

2) regex_class (included in the SPANG package)

Given a regular expression as a parameter, find classes with labels matching the pattern.

The target database is not limited to a specific one but arbitrary ones.

```
SELECT ?class ?label
WHERE {
   ?class a owl:Class ;
          rdfs:label ?label .
   FILTER regex(?label, "$1", "i")
}
```

3) mbgdl:get_ortholog (published at http://mbgd.genome.ad.jp/sparql/library/get_ortholog)

Given a UniProt ID as a parameter, retrieve its orthologs through the MBGD ortholog group.

The target database is MBGD.

```
SELECT ?uniprot
WHERE {
   ?group a orthology:OrthologsCluster ;
         orthology:hasHomologous/mbgd:gene/mbgd:uniprot uniprot:$1 ;
         orthology:hasHomologous/mbgd:gene/mbgd:uniprot ?uniprot ;
         void:inDataset mbgdr:default .
}
```

4) uniprot_xref (included in the SPANG package)

Given UniProt IDs from standard input and a database name as a parameter, retrieve their cross-references to the specified database.

The target database is UniProt.

```
SELECT ?uniprot ?xref
WHERE {
   VALUES (?uniprot) { $STDIN }
   ?uniprot rdfs:seeAlso ?xref .
   ?xref up:database <http://purl.uniprot.org/database/$1> .
}
```

5) diff_expr (included in the SPANG package)

Given a sample condition for expression profile, retrieve differentially expressed genes in the specified condition.

The target database Gene Expression Atlas.

```
SELECT ?uniprot ?probe ?experiment ?pubmed ?expr_label
WHERE {
   ?experiment atlas:pubmedid ?pubmed ;
         atlas:hasAnalysis/atlas:hasExpressionValue ?diff_expr .
   ?diff_expr rdfs:label ?expr_label ;
         atlas:hasFactorValue/a efo:$1 ;
         atlas:isMeasurementOf ?probe .
   ?probe atlas:dbXref ?uniprot .
   ?uniprot a atlas:UniprotDatabaseReference .
}
```

6) filter_by_go (included in the SPANG package)

Given UniProt IDs from standard input and a GO ID as a parameter, output proteins with the specified GO annotation.

The target database is UniProt.

```
SELECT ?uniprot
WHERE {
   VALUES (?uniprot) { $STDIN }
   ?uniprot up:classifiedWith obo:$1 .
}
```
