# Supplementary material for: SPANG: a SPARQL client supporting generation and reuse of queries for distributed RDF databases
Source: BMC Bioinformatics. 2017 Feb 8;18:93. doi: 10.1186/s12859-017-1531-1 (PMC5299738; doi:10.1186/s12859-017-1531-1)
Supplement: Additional file 3: — Example SPANG commands compared with the corresponding plain SPARQL queries. (PDF 70 kb) [file 12859_2017_1531_MOESM3_ESM.pdf]

Example SPANG commands compared with the corresponding plain SPARQL queries

1. Retrieve information about UniProt P02649.
2. Confine the results of query 1 to organism information.
3. Modify the query 2 to output the scientific name of the organism.
4. Retrieve annotation for UniProt P02649.
5. Search for classes matching a regular expression '^apolipoprotein'
6. Search MBGD for orthologs of UniProt K9Z723.
7. Search MBGD for orthologs of K9Z723, and then search UniProt for their annotations.
8. Search MBGD for orthologs of K9Z723, and then search UniProt for cross-references to PDB entries.
9. Search for classes matching a regular expression '^alzheimer'
10. Retrieve information about EFO_0000249 (Alzheimer's disease).
11. Retrieve differentially expressed genes in Alzheimer's disease, and then pick out proteins related to GO_0045202 (synapse).

	SPANG commands	Corresponding plain SPARQL queries	
1	spang uniprot -S uniprot:P02649	**Endpoint**	http://sparql.uniprot.org/sparql
		**Query**	PREFIX uniprot: <http://purl.uniprot.org/uniprot/><br><br>SELECT ?p ?o<br>WHERE {<br>uniprot:P02649 ?p ?o<br>}
2	spang uniprot -S uniprot:P02649 -P up:organism	**Endpoint**	http://sparql.uniprot.org/sparql
		**Query**	PREFIX uniprot: <http://purl.uniprot.org/uniprot/><br>PREFIX up: <http://purl.uniprot.org/core/><br><br>SELECT ?o<br>WHERE {<br>uniprot:P02649 up:organism ?o<br>}
3	spang uniprot -S uniprot:P02649 -P up:organism/up:scientificName	**Endpoint**	http://sparql.uniprot.org/sparql
		**Query**	PREFIX uniprot: <http://purl.uniprot.org/uniprot/><br>PREFIX up: <http://purl.uniprot.org/core/><br><br>SELECT ?o<br>WHERE {<br>uniprot:P02649 up:organism/up:scientificName ?o<br>}
4	spang uniprot uniprot_annot P02649	**Endpoint**	http://sparql.uniprot.org/sparql
		**Query**	PREFIX uniprot: <http://purl.uniprot.org/uniprot/><br>PREFIX up: <http://purl.uniprot.org/core/><br>PREFIX rdfs: <http://www.w3.org/2000/01/rdf-schema#><br>PREFIX skos: <http://www.w3.org/2004/02/skos/core#><br><br>SELECT ?gene ?organism ?function<br>WHERE {<br>uniprot:P02649 up:encodedBy/skos:prefLabel ?gene ;<br>up:organism/up:scientificName ?organism ;<br>up:annotation ?annot .<br>?annot a up:Function_Annotation ;<br>rdfs:comment ?function .<br>}
5	spang uniprot regex_class '^apolipoprotein'	**Endpoint**	http://sparql.uniprot.org/sparql
		**Query**	PREFIX owl: <http://www.w3.org/2002/07/owl#><br>PREFIX rdfs: <http://www.w3.org/2000/01/rdf-schema#><br><br>SELECT DISTINCT ?class ?label<br>WHERE {<br>?class a owl:Class ;<br>rdfs:label ?label .<br>FILTER regex(?label, "^apolipoprotein", "i")<br>}
6	spang mbgd mbgdl:get_ortholog K9Z723	**Endpoint**	http://sparql.nibb.ac.jp/sparql
		**Query**	PREFIX uniprot: <http://purl.uniprot.org/uniprot/><br>PREFIX void: <http://rdfs.org/ns/void#><br>PREFIX orthology: <http://purl.org/net/orth#><br>PREFIX mbgd: <http://purl.jp/bio/11/mbgd#><br>PREFIX mbgdr: <http://mbgd.genome.ad.jp/rdf/resource/><br><br>SELECT ?uniprot<br>WHERE {<br>?group a orthology:OrthologsCluster ;<br>orthology:hasHomologous/mbgd:gene/mbgd:uniprot uniprot:K9Z723 ;<br>orthology:hasHomologous/mbgd:gene/mbgd:uniprot ?uniprot ;<br>void:inDataset mbgdr:default .<br>}

7	spang mbgd mbgdl:get_ortholog K9Z723 | spang uniprot -S 1 -P rdfs:label	**Endpoint**	http://sparql.nibb.ac.jp/sparql
		**Query**	PREFIX uniprot: <http://purl.uniprot.org/uniprot/><br>PREFIX void: <http://rdfs.org/ns/void#><br>PREFIX orthology: <http://purl.org/net/orth#><br>PREFIX mbgd: <http://purl.jp/bio/11/mbgd#><br>PREFIX mbgdr: <http://mbgd.genome.ad.jp/rdf/resource/><br>PREFIX rdfs: <http://www.w3.org/2000/01/rdf-schema#><br><br>SELECT ?uniprot ?label<br>WHERE {<br>?group a orthology:OrthologsCluster ;<br>orthology:hasHomologous/mbgd:gene/mbgd:uniprot uniprot:K9Z723 ;<br>orthology:hasHomologous/mbgd:gene/mbgd:uniprot ?uniprot ;<br>void:inDataset mbgdr:default .<br>SERVICE <http://sparql.uniprot.org/sparql><br>{<br>?uniprot rdfs:label ?label .<br>}<br>}
8	spang mbgd mbgdl:get_ortholog K9Z723 | spang uniprot uniprot_xref PDB	**Endpoint**	http://sparql.nibb.ac.jp/sparql
		**Query**	PREFIX uniprot: <http://purl.uniprot.org/uniprot/><br>PREFIX void: <http://rdfs.org/ns/void#><br>PREFIX orthology: <http://purl.org/net/orth#><br>PREFIX mbgd: <http://purl.jp/bio/11/mbgd#><br>PREFIX mbgdr: <http://mbgd.genome.ad.jp/rdf/resource/><br>PREFIX rdfs: <http://www.w3.org/2000/01/rdf-schema#><br>PREFIX up: <http://purl.uniprot.org/core/><br><br>SELECT ?uniprot ?xref<br>WHERE {<br>?group a orthology:OrthologsCluster ;<br>orthology:hasHomologous/mbgd:gene/mbgd:uniprot uniprot:K9Z723 ;<br>orthology:hasHomologous/mbgd:gene/mbgd:uniprot ?uniprot ;<br>void:inDataset mbgdr:default .<br>SERVICE <http://sparql.uniprot.org/sparql><br>{<br>?uniprot rdfs:seeAlso ?xref .<br>?xref up:database <http://purl.uniprot.org/database/PDB> .<br>}<br>}
9	spang atlas regex_class ' ^alzheimer'	**Endpoint**	https://www.ebi.ac.uk/rdf/services/atlas/sparql
		**Query**	PREFIX owl: <http://www.w3.org/2002/07/owl#><br>PREFIX rdfs: <http://www.w3.org/2000/01/rdf-schema#><br><br>SELECT DISTINCT ?class ?label<br>WHERE {<br>?class a owl:Class ;<br>rdfs:label ?label .<br>FILTER regex(?label, "^alzheimer", "i")<br>}
10	spang atlas -S efo:EFO_0000249	**Endpoint**	https://www.ebi.ac.uk/rdf/services/atlas/sparql
		**Query**	PREFIX efo: <http://www.ebi.ac.uk/efo/><br><br>SELECT ?p ?o<br>WHERE {<br>efo:EFO_0000249 ?p ?o<br>}
11	spang atlas diff_expr EFO_0000249 > result	**Endpoint**	https://www.ebi.ac.uk/rdf/services/atlas/sparql
	cut -f1 result | spang uniprot filter_by_go GO_0045202	**Query**	PREFIX efo: <http://www.ebi.ac.uk/efo/><br>PREFIX atlas: <http://rdf.ebi.ac.uk/terms/atlas/><br>PREFIX rdfs: <http://www.w3.org/2000/01/rdf-schema#><br>PREFIX up: <http://purl.uniprot.org/core/><br>PREFIX obo: <http://purl.obolibrary.org/obo/><br><br>SELECT ?uniprot ?probe ?experiment ?pubmed ?expr_label<br>WHERE {<br>?experiment atlas:pubmedid ?pubmed ;<br>atlas:hasAnalysis/atlas:hasExpressionValue ?diff_expr .<br>?diff_expr rdfs:label ?expr_label ;<br>atlas:hasFactorValue/a efo:EFO_0000249 ;<br>atlas:isMeasurementOf ?probe .<br>?probe atlas:dbXref ?uniprot .<br>?uniprot a atlas:UniprotDatabaseReference .<br>SERVICE <http://sparql.uniprot.org/sparql><br>{<br>?uniprot up:classifiedWith obo:GO_0045202<br>}<br>}
